# Butyrate Stimulates Histone H3 Acetylation, 8-Isoprostane Production, RANKL Expression, and Regulated Osteoprotegerin Expression/Secretion in MG-63 Osteoblastic Cells

**DOI:** 10.3390/ijms19124071

**Published:** 2018-12-17

**Authors:** Mei-Chi Chang, Yunn-Jy Chen, Yun-Chia Lian, Bei-En Chang, Chih-Chia Huang, Wei-Ling Huang, Yu-Hwa Pan, Jiiang-Huei Jeng

**Affiliations:** 1 Chang Gung University of Science and Technology, Kwei-Shan, Taoyuan 333, Taiwan; mcchang@mail.cgust.edu.tw; 2 Department of Dentistry, Chang Gung Memorial Hospital, Taipei Branch, 6th Floor, 199, Tung-Hwa North Road, Taipei 105, Taiwan; yunyun445@cgmh.org.tw (Y.-C.L.); weiwei0110@hotmail.com (W.-L.H.); 3 School of Dentistry and Department of Dentistry, National Taiwan University Hospital and National Taiwan University Medical College, Taipei 100, Taiwan; chenyj@ntu.edu.tw; 4 Graduate Institute of Oral Biology, National Taiwan University Medical College, Taipei 100, Taiwan; beien@ntu.edu.tw; 5 Department of Dentistry, Cardinal Tien Hospital, New Taipei City 234, Taiwan; frank554@ms13.hinet.net; 6 Graduate Department of Craniofacial Dentistry, Chang-Gung University Medical College, Taoyuan 333, Taiwan

**Keywords:** bone resorption, butyric acid, HDAC inhibitor, osteoblasts, osteoprotegerin/RANKL, periodontal/root canal pathogens

## Abstract

Butyric acid as a histone deacetylase (HDAC) inhibitor is produced by a number of periodontal and root canal microorganisms (such as *Porphyromonas*, *Fusobacterium*, etc.). Butyric acid may affect the biological activities of periodontal/periapical cells such as osteoblasts, periodontal ligament cells, etc., and thus affect periodontal/periapical tissue destruction and healing. The purposes of this study were to study the toxic effects of butyrate on the matrix and mineralization marker expression in MG-63 osteoblasts. Cell viability was determined by 3-(4,5-dimethylthiazol-2-yl)-2,5-diphenyl tetrazolium bromide (MTT) assay. Cellular apoptosis and necrosis were analyzed by propidium iodide/annexin V flow cytometry. The protein and mRNA expression of osteoprotegerin (OPG) and receptor activator of nuclear factor kappa-B ligand (RANKL) were analyzed by Western blotting and reverse transcriptase-polymerase chain reaction (RT-PCR). OPG, soluble RANKL (sRANKL), 8-isoprostane, pro-collagen I, matrix metalloproteinase-2 (MMP-2), osteonectin (SPARC), osteocalcin and osteopontin (OPN) secretion into culture medium were measured by enzyme-linked immunosorbant assay. Alkaline phosphatase (ALP) activity was checked by ALP staining. Histone H3 acetylation levels were evaluated by immunofluorescent staining (IF) and Western blot. We found that butyrate activated the histone H3 acetylation of MG-63 cells. Exposure of MG-63 cells to butyrate partly decreased cell viability with no marked increase in apoptosis and necrosis. Twenty-four hours of exposure to butyrate stimulated RANKL protein expression, whereas it inhibited OPG protein expression. Butyrate also inhibited the secretion of OPG in MG-63 cells, whereas the sRANKL level was below the detection limit. However, 3 days of exposure to butyrate (1 to 8 mM) or other HDAC inhibitors such as phenylbutyrate, valproic acid and trichostatin stimulated OPG secretion. Butyrate stimulated 8-isoprostane, MMP-2 and OPN secretion, but not procollagen I, or osteocalcin in MG-63 cells. Exposure to butyrate (2–4 mM) for 3 days markedly stimulated osteonectin secretion and ALP activity. In conclusion, higher concentrations of butyric acid generated by periodontal and root canal microorganisms may potentially induce bone destruction and impair bone repair by the alteration of OPG/RANKL expression/secretion, 8-isoprostane, MMP-2 and OPN secretion, and affect cell viability. However, lower concentrations of butyrate (1–4 mM) may stimulate ALP, osteonectin and OPG. These effects are possibly related to increased histone acetylation. These events are important in the pathogenesis and repair of periodontal and periapical destruction.

## 1. Introduction

Butyrate is a well-known histone deacetylase (HDAC) inhibitor [1]. Butyric acid can be generated by microorganisms present in the dental plaque and biofilms of the root surface and root canals, and is involved in the pathogenesis of periodontitis and apical periodontitis. Periodontal and root canal (dental pulp) pathogens, such as *Porphyromonas gingivalis* (*P*. *gingivalis*) and other microorganisms, are widely distributed in the periodontal pocket, root canal and apical lesion of the root apex [2,3,4,5,6,7]. These pathogenic microorganisms are able to attack and invade adjacent tissues or release toxic metabolites such as endotoxin, proteases and short chain fatty acids (e.g., propionic acid, and butyric acid) which affect cellular responses [6,7,8]. 

Recent studies have found that butyrate plays an important role in periodontal and apical lesions in the gums and periodontal tissues. The concentrations of butyric acid may reach 0.2–16 mM in the gingival sulcus [9,10], and the concentration of butyric acid in root canals decreases after endodontic treatment [11]. Butyric acid may affect the biological activities of periodontal/periapical cells such as osteoblasts, periodontal ligament cells, gingival fibroblasts (GF) and inflammatory cells, etc., [12,13,14], thus affecting periodontal/periapical tissue destruction and healing. 

Bone homeostasis depends on the balance of bone resorption by osteoclasts and formation by osteoblasts. An imbalance of bone turnover may cause diseases such as osteoporosis, bone resorption, periodontitis, etc. [15]. Interactions between osteoblasts and osteoclasts via direct contact or cytokine release is important for bone homeostasis. Osteoblasts may affect osteoclast activity through the osteoprotegerin (OPG)/receptor activator of nuclear factor kappa-B (RANK)/RANK ligand (RANKL) system [15]. An increase in RANK, RANKL and OPG expression in the progression of periodontitis and apical periodontitis has been reported [16,17,18]. An increased ratio of RANKL/OPG can be a marker showing the presence of periodontitis and bone resorption [18]. These changes in expression of OPG and RANKL during periodontitis may be related to the presence of various periodontal and root canal pathogens [19,20] and related toxins. *P. gingivalis* lipopolysaccharide (LPS) has been shown to affect RANKL and RANKL/OPG ratios of periodontal ligament fibroblasts [21]. However, limited information is known about butyric acid on the OPG and RANKL expression of osteoblastic cells and their possible contribution to periodontal bone destruction.

Previously, we have found that butyrate suppresses adhesion, cell growth and protein synthesis, but induces reactive oxygen species and the cell cycle arrest of gingival fibroblasts (GF) [22,12]. It also inhibited cell growth, collagen I expression, and induced cell cycle arrest and the p21 and p27 expression of osteoblasts [23]. The purposes of this study was to further study the effects of butyrate on bone metabolism and turnover mediated by the alteration of OPG/RANKL mechanisms and other matrix/mineralization molecules. Understanding these effects and mechanisms can help us with the treatment and prevention of subsequent periodontal and periapical diseases.

## 2. Results

### 2.1. Stimulation of Histone H3 Acetylation by Butyrate

**Figure 1:**
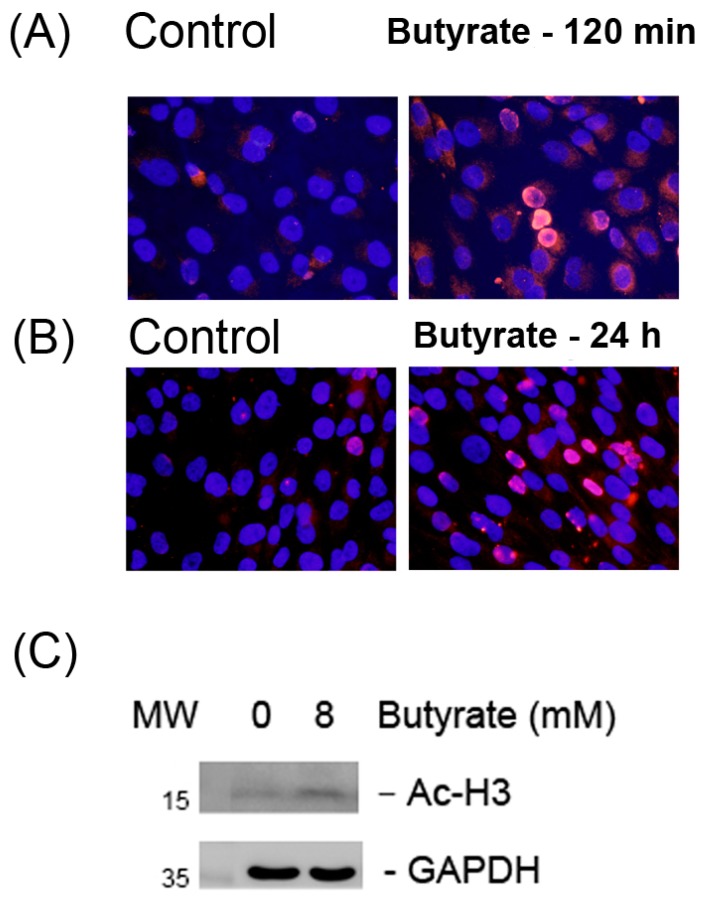
The stimulation of the histone H3 acetylation of MG63 cells as analyzed by immunofluorescent staining (IF) and Western blotting. (**A**) IF pictures of Ac-H3 expression: Control (120 min) and butyrate (8 mM)-treated MG-63 cells for 120 min. (**B**) IF pictures of Ac-H3 expression: Control (24 h) and butyrate (8 mM)-treated MG63 cells for 24 h, 400×, original magnification, (**C**) Western blotting of control and 8 mM butyrate-treated MG-63 cells for 24 h. One representative IF study result was shown. GADPH: Glyceroaldehyde-3-dehydrogenase. MW: Molecular weight (KD).

Control MG63 cells showed limited nuclear staining of Ac-H3 (Figure 1A). Butyrate (8 mM) stimulated the histone H3 acetylation of MG-63 cells as analyzed by immunofluorescent (IF) staining. An increase in the red fluorescence of nuclear staining of MG-63 cells was noted after 120 min of exposure to 8 mM butyrate (Figure 1A). An increase in Ac-H3 nuclear staining was also noted when MG-63 cells were exposed to butyrate for 24 h (Figure 1B). Accordingly, butyrate stimulated the Ac-H3 expression of MG-63 cells as analyzed by Western blotting (Figure 1C).

### 2.2. Morphology of MG-63 Cells after Exposure to Butyrate for Three Days

**Figure 2:**
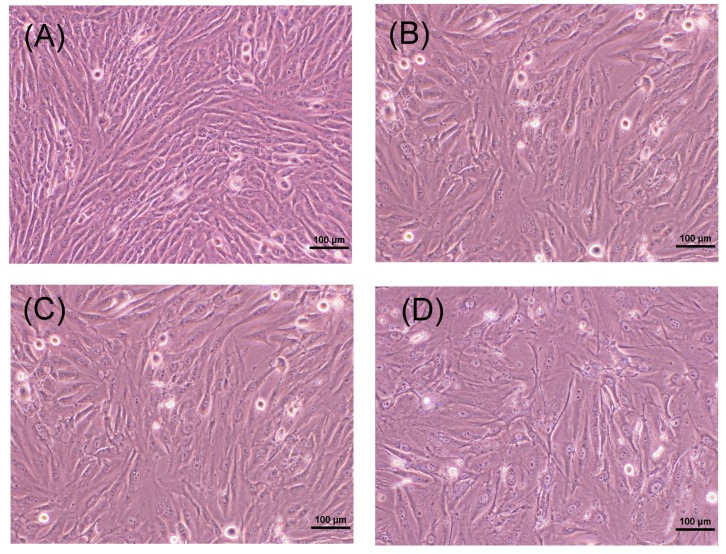
Morphologic changes of MG-63 cells (10^4^ cells/well) after exposure to different concentrations of butyrate for three days. (**A**) Control, (**B**) 4 mM butyrate, (**C**) 8 mM butyrate, (**D**) 16 mM butyrate. 100× original magnification (bar = 100 μm). One representative result was shown.

When non-confluent MG-63 cells (1 × 10^4^ cells/well) were cultured for three days, cells grew to confluence. MG-63 cells were fibroblast-like in appearance (Figure 2A). When exposed to butyrate (4 and 8 mM) for three days, the cell density of MG-63 cells slightly decreased (Figure 2B,C). Exposure to 16 mM for three days further decreased the cell density, with spaces between cells suggesting an increasing toxicity of butyrate (Figure 2D). 

### 2.3. Effect of Butyrate on the Growth and Cell Viability of MG-63 Cells

**Figure 3:**
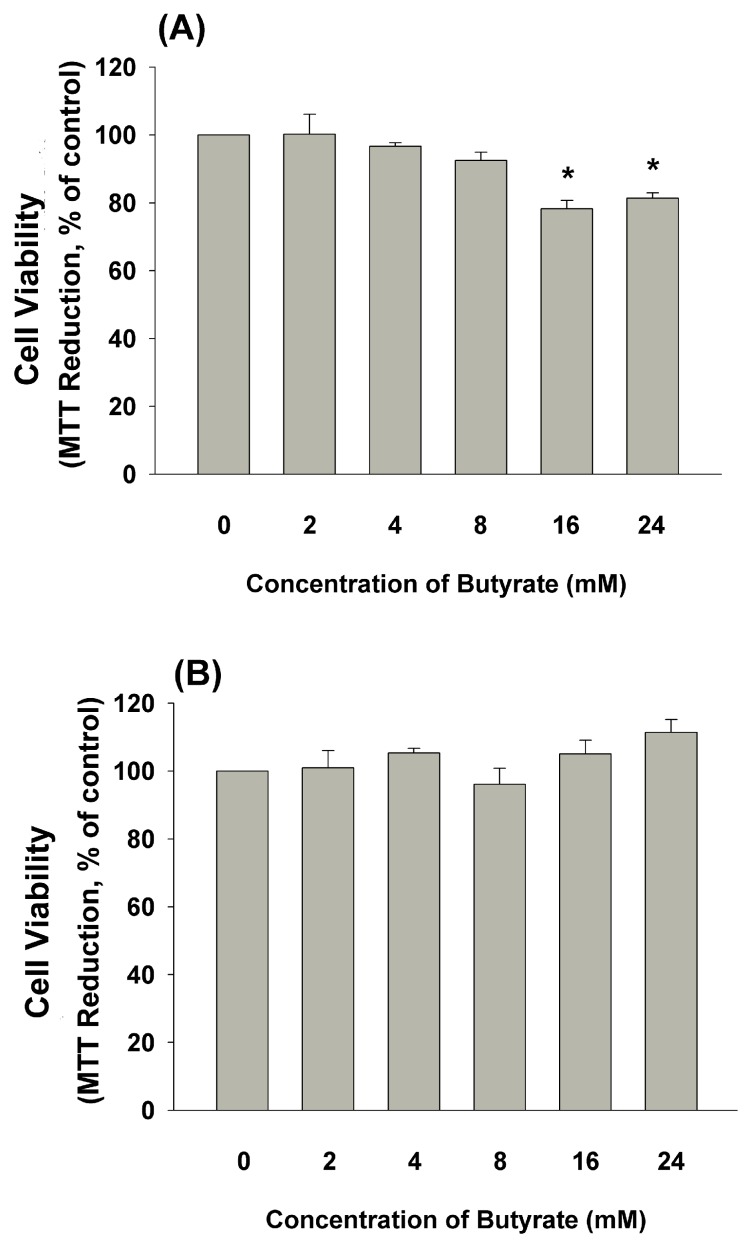
Effect of butyrate on the cell viability of MG63 cells: (**A**) MG63 cells (1 × 10,000 cells/24-well) were exposed to butyrate for 3 days, (**B**) roughly confluent MG63 cells (1 × 100,000 cells/24-well) were exposed to butyrate for three days. Cell viability was determined by 3-(4,5-dimethylthiazol-2-yl)-2,5-diphenyl tetrazolium bromide (MTT) assay. Results were expressed as a percentage of control (Mean ± SE). Statistically significant difference when compared with the control (*p* < 0.05) denoted by *.

Accordingly, when non-confluent MG-63 cells (1 × 10^4^ cells/well) were exposed to butyrate (16 and 24 mM) for three days, cell viability decreased (Figure 3A). On the other hand, when confluent MG-63 cells (1 × 10^5^ cells/well) were exposed to butyrate for three days, cell viability showed no marked difference (Figure 3B).

### 2.4. Effect of Butyrate on the Apoptosis and Necrosis of MG-63 Cells

**Figure 4:**
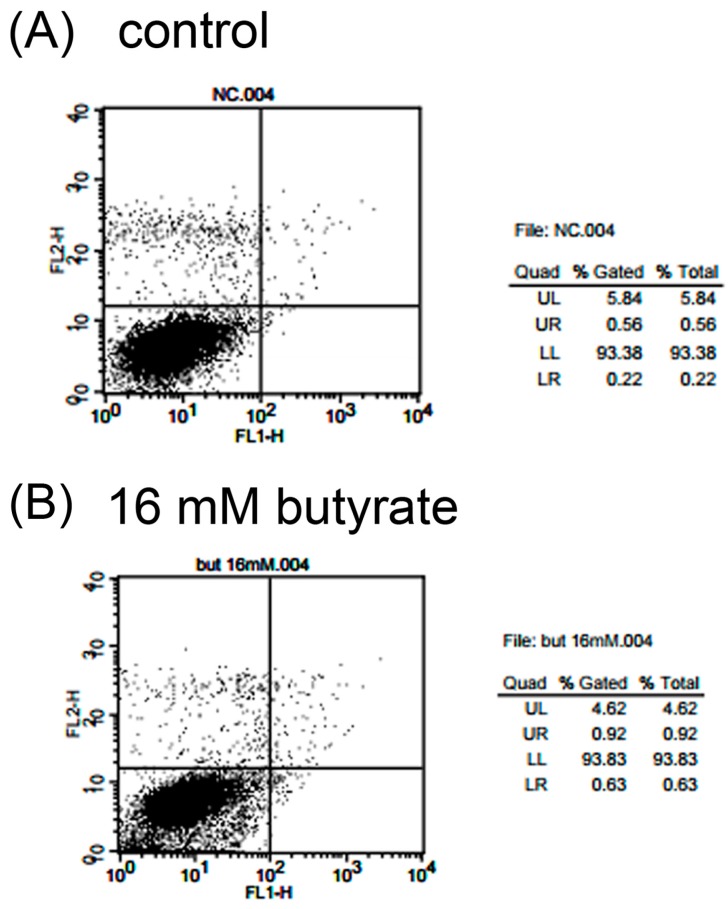
Effect of butyrate on the induction of the apoptosis and necrosis of MG63 cells as analyzed by propidium iodide (PI) + annexin V flow cytometry. UL (upper left): Necrosis, UR (upper right) and LR (lower right): Apoptosis. One representative PI and annexin V flow cytometry histogram was shown. (**A**) Control and (**B**) 16 mM butyrate-treated cells.

Propidium iodide (PI) and annexin V flow cytometric analysis was used to determine the induction of apoptosis and necrosis of MG-63 cells after exposure to various concentrations of butyrate. As shown in Figure 4A, exposure to 16 mM butyrate could not evidently induce apoptosis (upper right (UR) & lower right (LR)) and necrosis (upper left (UL)) of MG-63 cells. Quantitatively, the percentage of cells (%) residing in the UL (necrotic cells) increased from 4.19% to 4.79% after exposure to 24 mM butyrate. In addition, the percentage of cells in the UR (apoptotic cells) and LR (pro-apoptotic cells) quadrants changed from 0.85% and 0.41% in the control to 1.28% and 1.05%, respectively, with 16 mM butyrate (Figure 4B, Table 1).

### 2.5. Effect of Butyrate on OPG and RANKL mRNA and Protein Expression

**Figure 5:**
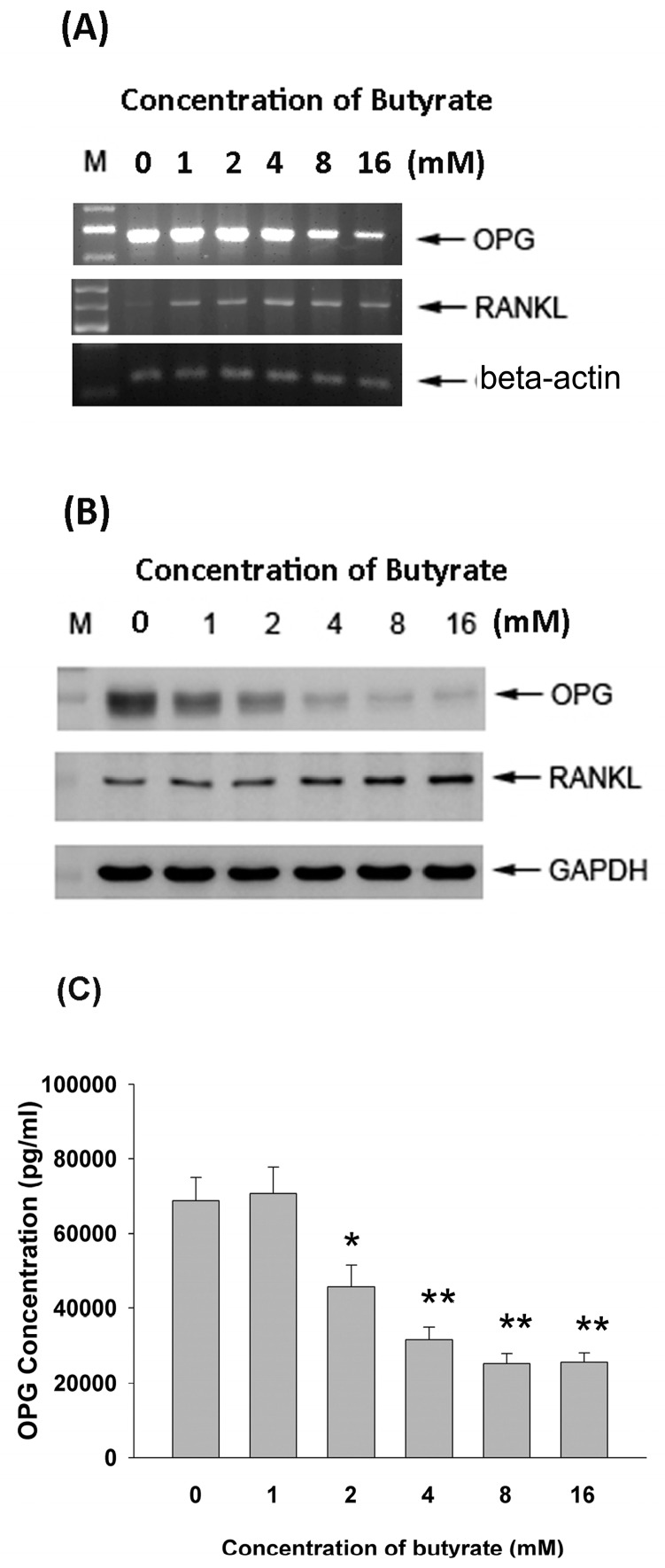
Effect of 24-h exposure to butyrate on the osteoprotegerin (OPG) and receptor activator of nuclear factor kappa-B ligand (RANKL) expression of MG63 osteoblastic cells. (**A**) RT-PCR analysis of mRNA expression, (**B**) Western blot analysis of OPG and RANKL protein expression, (**C**) ELISA analysis of OPG level in culture medium. Statistically significant differences are denoted by * and ** (*p* < 0.05 and *p* < 0.01) when compared with the control, respectively.

The exposure of MG-63 cells to butyrate for 24 h stimulated RANKL mRNA expression (>1 mM), whereas it inhibited OPG mRNA expression as analyzed by RT-PCR (Figure 5A), and thus decreased the ratio of OPG/RANKL. Butyrate also stimulated RANKL protein expression (>4 mM). On the contrary, it inhibited OPG protein expression (>1 mM) as analyzed by Western blotting (Figure 5B).

### 2.6. Effect of Butyrate on OPG and RANKL Secretion of MG-63 Cells

Since OPG and RANKL are important for the regulation of bone resorption, we further determined whether the exposure of MG-63 cells to butyrate may affect the secretion of OPG and RANKL. Interestingly, butyrate markedly inhibited the secretion of OPG in MG-63 cells at concentrations ranging from 2–16 mM after 24 h of exposure (Figure 5C). Unexpectedly, the sRANKL level in the culture medium was below the detection limit (data not shown).

### 2.7. Effect of 3 days of Exposure of MG-63 Cells to Butyrate or other HDAC Inhibitors on OPG Secretion

**Figure 6:**
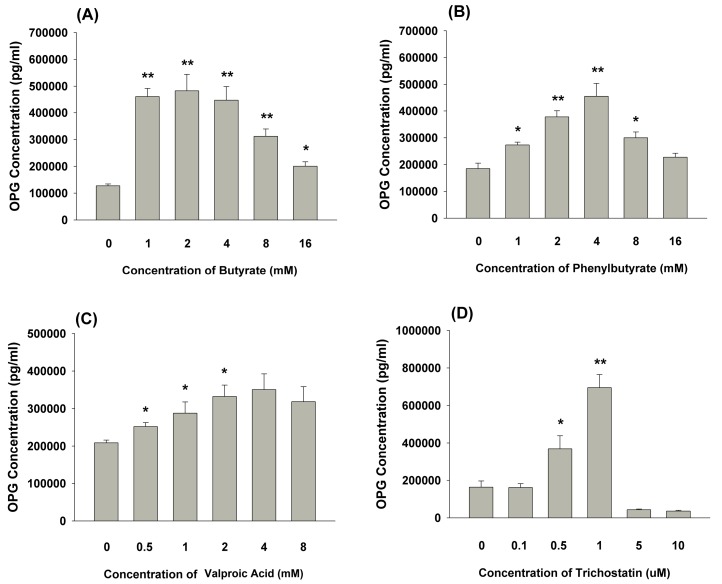
OPG secretion of MG-63 cells after three days of exposure to (**A**) butyrate, (**B**) phenylbutyrate, (**C**) valproic acid, (**D**) trichostatin for three days. The OPG level in the culture medium was determined by ELISA and expressed as mean ± SE (pg/mL). Statistically significant differences are denoted by * and ** (*p* < 0.05 and *p* < 0.01) when compared with the control, respectively.

The exposure of MG-63 cells to butyrate for 3 days stimulated OPG secretion, with a maximal stimulation at about 1 to 4 mM (Figure 6A). Similarly, phenylbutyrate (1 to 8 mM) as an HDAC inhibitor also stimulated OPG secretion. However, this effect became less evident at 16 mM, possibly due to toxicity (Figure 6B). Valproic acid as the other HDAC inhibitor also induced the OPG production of MG-63 cells at concentrations ranging from 0.5 to 2 mM (*p* < 0.05) or higher (4 mM, *p* = 0.06) (Figure 6C). Moreover, trichostatin also stimulated the OPG secretion of MG-63 cells at concentrations of 0.5 and 1 μM. However, the marked suppression of OPG secretion of MG-63 cells by 5 and 10 μM of trichostatin was noted, possibly due to cytotoxicity (Figure 6D).

### 2.8. Effect of Butyrate on 8-Isoprostane, Pro-Collagen I, MMP-2, Osteopontin, Osteonectin, and Osteocalcin Secretion as well as the ALP Activity of MG-63 Cells

**Figure 7:**
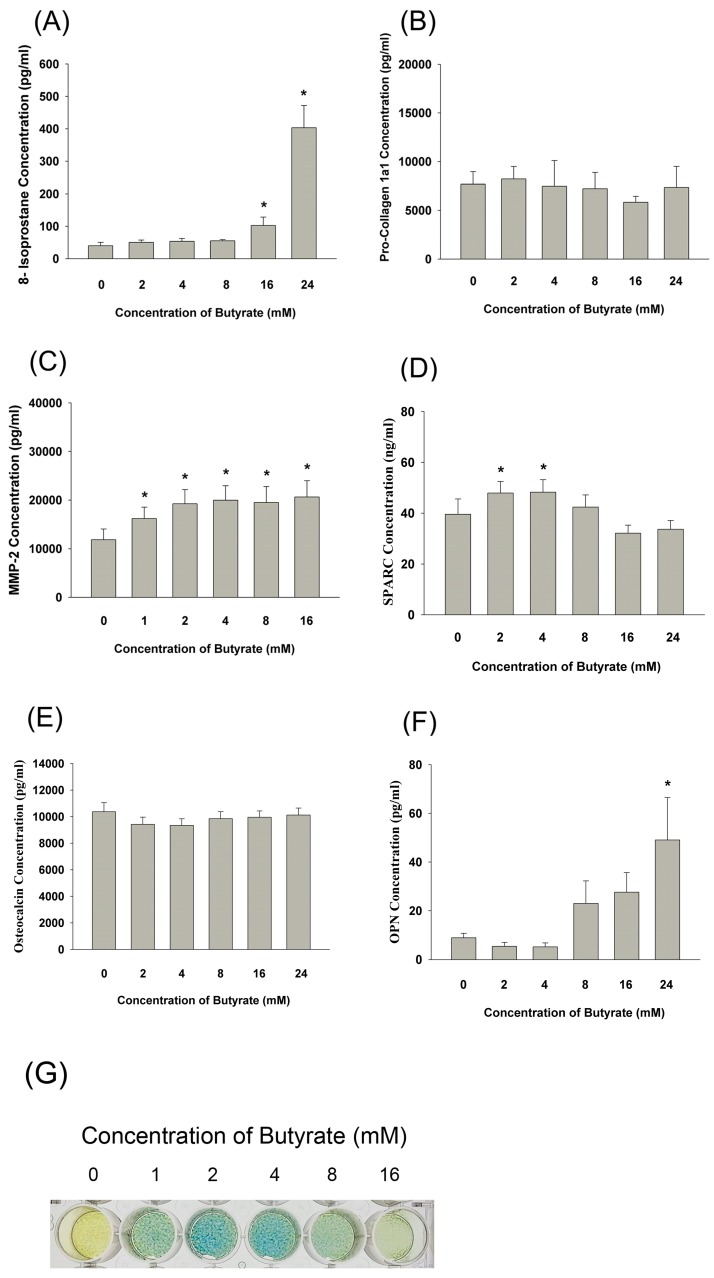
Effect of three days of exposure to butyrate on (**A**) 8-isoprostane, (**B**) pro-collagen I, (**C**) MMP-2, (**D**) osteonectin, (**E**) osteocalcin, and (**F**) osteopontin (OPN) secretion of MG-63 cells as analyzed by ELISA. Results were expressed as mean ± SE. Statistically significant differences are denoted by * when compared with the control (*p* < 0.05). (**G**) Effect of butyrate on the alkaline phosphatase (ALP) activity of MG-63 cells. One representative ALP staining picture was shown.

Exposure to butyrate significantly stimulated the 8-isoprostane production of MG63 cells (Figure 7A). For matrix turnover, butyrate showed no marked effect on pro-collagen 1a1 secretion after 24 h of exposure (Figure 7B). On the other hand, butyrate (>1 mM) induced the MMP-2 secretion of MG-63 cells (Figure 7C), and butyrate (2 to 4 mM) showed a mild stimulatory effect on osteonectin (SPARC) secretion (Figure 7D). For mineralization markers, butyrate showed little stimulatory effect on the osteocalcin production of MG-63 cells (Figure 7E). Moreover, butyrate (>24 mM) evidently stimulated the osteopontin (OPN) secretion of MG63 cells (Figure 7F). Interestingly, butyrate stimulated the ALP activity of MG-63 cells, with maximal stimulation at about 2 to 4 mM as revealed by ALP staining (Figure 7G).

## 3. Discussion

Butyrate is a well-known histone deacetylase (HDAC) inhibitor produced by microorganisms of the oral cavity and intestinal tract. Short chain fatty acids (SCFA) including propionic acid and butyric acid may contribute to periodontal/periapical tissue toxicity [3,14]. The levels of SCFA may reach 0.2–16 mM in gingival crevicular fluid (GCF), and periodontal treatment decreases the concentrations of SCFA in GCF [9,10,14], suggesting the important role of SCFA in periapical/periodontal diseases. Possibly, SCFA may induce an inflammatory response and affect the viability and biological activities of periodontal and periapical tissues, leading to bone destruction. In this study, butyrate inhibited cell viability, but showed no appreciable cytotoxicity to near-confluent MG-63 cells, indicating that the presence of higher concentrations of butyrate in the periodontal pocket or root canal may potentially impair the clinical tissue healing response. Similarly, butyrate suppressed the adhesion, growth and protein synthesis of gingival epithelial cells and fibroblasts. It also induced the death and cytokine release of inflammatory cells [9,12,14,22]. However, the inhibition of cell viability by butyrate is not mediated by apoptotic and necrotic cell death. Butyrate induces a minimal apoptotic and necrotic effect on MG-63 cells as analyzed by propidium iodide (PI) and annexin V flow cytometry. Other studies have reported the induction of apoptosis on colon cancer cells, B cells and other kinds of cells by butyrate [1,24,25], showing the effect of butyrate may vary by cell type. Ho & Chang (2007) further discovered the toxicity of butyrate on dental pulp cells and its relation to glutathione (GSH) depletion [26]. Interestingly, butyrate showed a possible toxicity for proliferating MG-63 cells, which increases proportionally with the concentration of butyrate. The actual reasons and meanings for different results with butyrate are unclear and await further investigation. 

SCFAs have inflammatory and anti-inflammatory effects depending on the cells and conditions [27,28]. As an HDAC inhibitor, butyrate may stimulate histone acetylation [1]. Interestingly, butyrate is shown to attenuate the tumor necrosis factor-α (TNF-α and LPS-induced interleukin-6 (IL-6) expression of endothelial cells via the activation of GPR41/GPR43 receptors. However, the prevention of TNF-α and LPS-induced IL-8 expression of endothelial cells by butyrate is related to its inhibition of histone deacetylase (HDAC), but not its activation of GPR receptors [29]. Butyrate stimulates the differentiation of mesenchymal stem cells to smooth muscle cells or osteoblasts by enhancing histone H3 and H4 acetylation, and the down-regulation of HDAC2 or MEK/ERK-Runx2 signaling [30,31]. In this study, butyrate also induced the histone H3 acetylation of MG-63 osteoblasts, implicating the possible involvement of histone acetylation by butyrate in periodontal destruction and healing. Recently, the stimulation of histone acetylation by HDAC inhibitors such as trichostatin A, butyrate, and valproic acid has been shown to promote osteoblast maturation and bone formation [32]. During osteoblast differentiation, histones H3 and H4 are hyperacetylated. The knockdown of HDAC1 by siRNA also promotes osteoblast differentiation [33]. These results suggest the possible effect of butyrate on bone turnover.

Previous study has found butyrate causes the stimulation of the reactive oxygen species (ROS) production of MG-63 cells [23]. 8-isoprostane (8-iso-prostaglandin F2α), as an oxidative stress marker and lipid peroxidation product, is generated by the free-radical catalyzed peroxidation of essential fatty acids such as arachidonic acid to stimulate redox-sensitive signaling pathways and transcriptional factors [34,35,36]. Isoprostanes may mediate vasoconstriction, tissue inflammation, perception of pain, vascular reperfusion, paracetamol poisoning, liver cirrhosis, atherosclerosis and cancer [34,35,37]. Limited information is known about the generation of isoprostanes by osteoblasts and their roles in bone turnover. In this study, 8-isoprostane as an oxidative stress marker was also found to be stimulated in MG-63 cells by butyrate, suggesting the induction of lipid peroxidation by butyrate. This may partly explain the elevated salivary, GCF or root canal 8-isoprostane level in patients with periodontitis, pulpitis or chronic apical periodontitis [38,39,40,41], suggesting the involvement of increased oxidative stress in pulpal/periapical pain, periodontal and periapical bone destruction. It has been found that iso-PGE_2_ but not iso-PGF_2_ alpha had an inhibitory effect on the induction of alkaline phosphatase activity in MC3T3-E1 preosteoblasts [42]. Urinary F2-isoprostanes levels have been found to show a negative correlation with bone mineral content and bone mineral density [43]. It has been found that patients with diabetes, obesity, hypercholesterolemia, smokers, etc., have higher levels of urinary isoprostane [44]. Possibly, urinary isoprostane levels can be used as marker of periodontal and periapical diseases in the future. An increase of 8-isoprostane by butyrate may be involved in the pathogenesis of periodontal/periapical diseases.

Bone turnover is tightly regulated by the interaction of osteoblasts and osteoclasts [15], possibly via the OPG/RANKL system. It has been shown that periodontal/root canal pathogens may stimulate the RANKL expression of bone marrow cells [19,20]. Injection of anti-RANKL and osteoprotegerin fusion protein into rat gingiva may attenuate the bone resorption induced by *P. gingivalis* infection [20]. *P. gingivalis* LPS is shown to stimulate RANKL and increase the RANKL/OPG ratio in periodontal ligament fibroblasts [21]. An increased expression of RANK, RANKL and OPG during the progression of periodontitis and apical periodontitis has been reported [16]. Belibasakis et al. (2013) also found the protective role of OPG against root resorption and the increased apical RANKL/OPG ratio in bone resorption [17]. The level of RANKL is found to be increased, but the OPG level is decreased, in severe periodontitis [45]. Cyst and granuloma tissues from periapical lesions also show an increased expression of RANKL and the importance of RANKL and OPG products in apical bone destruction [17,46,47,48]. However, butyrate is shown to stimulate OPG expression in normal human osteoblasts [49]. In this study, butyrate was shown to stimulate RANKL, but decrease OPG expression and the secretion of osteoblasts, within 24 h of exposure. This may partly explain why butyric acid may be involved in periodontal and periapical bone destruction and turnover. However, exposure to lower concentrations of butyrate (<8 mM) for three days was shown to stimulate OPG secretion, suggesting that exposure time and concentrations of butyric acid in the gingival sulcus and apical region may affect the tissue healing responses. Moreover, phenylbutyrate, valproic acid and trichostatin, three HDAC inhibitors, also stimulated the OPG secretion of MG-63 cells, showing the possible involvement of histone acetylation in this event.

While mesenchymal stem cells are known to become flattened, spread and to differentiate into osteoblasts [50], butyrate is shown to affect the differentiation of colon cancer cells and osteoblastic cells [1,24,49,51]. Butyrate (0.1 mM) stimulates the expression of mineralization markers in periodontal ligament cells, whereas butyrate exhibits cytotoxicity at concentrations higher than 1 mM [52]. Butyrate (0.1 mM) stimulates calcium content, mineralized nodule formation and the expression of bone sialoprotein (BSP), osteopontin (OPN) and OPG, but showed little effect on proliferation, M-CSF, type I collagen expression and the ALP activity of normal human osteoblasts [49]. It seems that butyrate mainly affects osteoblasts in the late stage of differentiation rather than osteoblasts at the early stage. It also stimulates ALP activity in MC3T3 osteoblasts [51]. Butyrate stimulates bone sialoprotein (BSP) expression in rat ROS17/2.8 osteoblast-like cells [1]. BSP may function in the initial mineralization stage of bone and is crucial for osteoblast differentiation, bone matrix mineralization and tumor metastasis [1]. On the contrary, butyrate (1 mM) suppressed the expression of Runx2, Osterix, Dlx5, Msx2, osteocalcin, ALP and BSP expression as well as mineralized nodule formation in ROS17/2.8 osteoblasts [13]. Similarly, butyrate (0.1–1 mM) also stimulates cyclooxygenase-2 (COX-2), collagen, OPN, EP1, and EP2 prostaglandin E2 receptor expression and PGE2 production, but showed little effect on the proliferation of ROS17/2.8 cells [53]. In this study, butyrate stimulated MMP-2 and OPN at higher concentrations, but showed no marked stimulatory effect on the pro-collagen I and osteocalcin (SPARC) of MG63 osteoblastic cells. At concentrations of 1–4 mM, it may stimulate ALP activity and osteonectin secretion. These results suggest that butyrate may affect the matrix turnover and differentiation of osteoblastic cells. Given the wide heterogeneity, the differential results of butyrate may be related to differences in cell types, such as primary cultures of rats, other cells from murine bone tissue, other cells from immortalized cultures, osteosarcoma cells and also those from clinical studies, etc. Other factors including differentiation status (pre-osteoblasts and osteoblasts), butyrate concentration, exposure time, and cell density may also affect the results and clinical responses of apical/periodontal tissues to butyric acid. 

We have previously found that propionate and butyrate may affect the activities including the growth, attachment, migration, reactive oxygen species and cell cycle alteration of gingival fibroblasts and osteoblasts [12,22,23]. In conclusion, the exposure of MG-63 osteoblastic cells to butyrate leads to histone H3 acetylation and 8-isoprostance production. Butyrate inhibited cell viability but did not induce the apoptosis of MG-63 osteoblasts. Higher concentrations of butyrate further stimulate MMP-2, OPN and RANKL but inhibit OPG expression and secretion, affecting the matrix turnover and mineralization of bone tissues. Lower concentrations of butyrate may stimulate ALP and osteonectin. These results may increase our understanding of the role of butyric acid in the pathogenesis of apical and periodontal bone destruction and can be helpful for disease prevention and treatment in the future. 

## 4. Materials and Methods

### 4.1. Materials 

Dulbecco’s modified Eagle’s medium (DMEM), fetal bovine serum (FBS), trypsin/EDTA, and penicillin/streptomycin were from Gibco (Life Technologies, Grand Island, NY, USA). Antibodies of glyceroaldehyde-3-dehydrogenase (GAPDH) were purchased from Santa Cruz, whereas antibodies for Ac-H3, OPG and RANKL were from GeneTex. Ethidium bromide, agarose and kits for reverse transcription (RT) and polymerase chain reaction (PCR) were purchased from HT Inc., UK. Total RNA isolation kits were from Qiagen Inc. (Santa Clarita, CA, USA). Specific PCR primer sets were synthesized by Genemed Biotechnologies, Inc. (San Francisco, CA, USA). Protein assay kits were obtained from Bio-Rad (Bio-Rad Labs, Hercules, CA, USA). Sodium butyrate was obtained from Sigma (Sigma-Aldrich Company, St. Louis, MO, USA). MG-63 cells were obtained from the American Type Culture Collection (ATCC). OPG, RANKL, pro-collagen I, MMP-2, osteopontin, osteocalcin and osteonectin (SPARC) ELISA kits were from R&D. 8-Isoprostane ELISA kits were from Cayman.

### 4.2. Immunofluorescent Staining of Ac-H3 Expression

MG-63 cells (1 × 10^5^) were seeded onto a 24-well culture with sterile coverslips in 1 mL DMEM with 10% FBS. After 24 h, a culture medium containing various concentrations of butyrate (1-16 mM) was added and cells were further incubated for 24 h. MG-63 cells were also exposed to 8 mM butyrate for different time points (5–120 min). The medium was removed, cells were washed with PBS and fixed in 4% paraformaldehyde for 20 min. Cells were further washed with PBS, permeabilized with 2% Triton X-100, and exposed to 0.3% *v*/*v* H_2_O_2_ for 20 min. After rinsing with PBS, 5% bovine serum albumin (BSA) was used for blocking the nonspecific sites for 1 h, and then cells were incubated with primary antibodies (acetyl-histone H3) (1:1000, *v*/*v*, as 0.1 µg/mL) at room temperature overnight. Following washing by PBS, cells were incubated in tetramethylrhodamine (TRITC)-conjugated secondary antibodies in the dark for 1 h and the nucleus was counterstained with DAPI (1:1000, at 5 μg/mL) for 30 min. Finally, the cells on coverslips were mounted and photographed/observed under an inverted microscope and DP Controller/Manager software (Olympus IX71, Olympus Corporation, Tokyo, Japan) [23,54]. 

### 4.3. Effect of Butyrate on the Proliferation and Viability of MG-63 Cells 

Briefly, cells were plated into 24-well culture plates at a density of 1 × 10^4^ or 1 × 10^5^ cells/well. Cells were exposed to fresh medium containing various concentrations of butyrate (2, 4, 8, 16 and 24 mM) for 24 h or 3 days. The morphology of cells was taken under a microscope. Cell viability was estimated by MTT assay as before [22,23]. 

### 4.4. Inducing the Apoptosis and Necrosis of MG-63 Cells by Butyrate: PI and Annexin V Flow Cytometric Analysis

Briefly, 5 × 10^5^ cells in a 6-well culture plate were exposed to 2 mL fresh medium containing different concentrations of butyrate (2, 4, 8, 16 and 24 mM) for 24 h. Annexin V/PI dual staining flow cytometric analysis was conducted as previously [55,56]. In short, both the floating and attached cells were harvested. Cells were then washed with PBS, resuspended in 400 µL (4-(2-hydroxyethyl)-1-piperazineethanesulfonic acid) and (HEPES, 10 mM-NaOH, pH 7.4, 140 mM NaCl, 2.5 mM CaCl_2_) solution, the annexin V-fluorescein isothiocyanate (FITC, Becton Dickson, Franklin Lakes, NJ, USA))/ PI (50 μg/mL) staining solution was added, and the cells were incubated in the dark for 30 min. The annexin V-FITC and PI fluorescence of cells were measured by FACSCalibur Flow Cytometry (Becton Dickinson) immediately after. For the analysis of each sample, 15,000 events were recorded. The fluorescence of the cells of samples was gated and counted. The percentage of cell localization in the upper right (apoptotic), upper left (necrotic cells), lower right (pro-apoptotic cells), and lower left (control) portion of the histogram was determined for comparison.

### 4.5. Effect of Butyrate on the mRNA Expression of OPG, RANKL of Cultured MG-63 Cells

About 1 × 10^6^ MG-63 cells in 10 cm culture dishes were exposed in fresh medium containing butyrate (1, 2, 4, 8, 16 mM). Total RNA was isolated using Qiagen RNA isolation kits. Semi-quantitative reverse-transcriptase and polymerase chain reaction (RT-PCR) was performed as follows: In brief, 3 μg of denatured total RNA was reverse-transcribed in a total volume of 44.5 μL reaction mixture containing 4 μL of random primer (500 μg/mL), 8 μL of dNTP (2.5 mM), 4.5 μL of 10x RT buffer, 1 μL of RNase inhibitor (40 U/μL) and 0.5 μL of RT (21 U/μL) at 42 °C for 90 minutes. Four microliters of cDNA were then used for PCR amplification in a reaction volume of 50 μL containing 5 μL of 10× Super TAQ buffer, 4 μL of dNTP (2.5 mM), 1 μL of each specific primer, and 0.2 μL of Super TAQ enzyme (2 U/μL). The reaction mixture was initially heated to 94 °C for 5 minutes in the first cycle, then the reaction was amplified for 15–35 cycles of 94 °C for 30 s, 55 °C for 30 s and then 72 °C for 30 s with a thermal cycler (Perkin Elmer 4800, PE Applied Biosystems, Foster city, CA, USA). Finally, the reaction was set at 72 °C for a further 10 min. 

Specific primer sets for OPG—TCAAGCAGGAGTGCAATCG and AGAATGCCTCCTCACACAGG—and RANKL—CCAGCATCAAAATCCCAAGT and CCCCTTCAGATGATCCTTC—with PCR amplified products of 342 and 603 base pairs, respectively, were used [57]. An expression of beta-actin was used as a control [58]. The amplified DNA products are loaded onto 1.8% of agarose gel in 1× of Tris/Borate/EDTA (TBE) buffer for electrophoresis. Gels were stained with ethidium bromide and photographs were taken. The range of amplified DNA product that was linear in relation to the input RNA was used for data presentation. The amplification of the BAC gene is used as a control. 

### 4.6. Effects of Butyrate on OPG, RANKL and Ac-H3 Protein Expression of MG-63 Cells 

MG-63 cells (1 × 10^6^ cells) in 10-cm culture dishes were exposed to fresh medium containing various concentrations of butyrate (1, 2, 4, 8, 16 mM) for 24 hrs. Cell lysates were collected as described previously using freshly prepared lysis buffer (10 mM Tris-HCl, pH 7; 140 mM sodium chloride; 3 mM magnesium chloride; 0.5% NP-40; 2 mM phenylmethylsulfonyl fluoride; 1% aprotinin; and 5 mM dithiothreitol) [12,58,59,60]. The protein concentrations of the cell lysates were measured by Bio-Rad protein assay kits. Equal amounts of protein (50 µg/lane) were separated by 12% SDS-polyacrylamide gel electrophoresis (Scie-Plas, Cambridge, UK) and transferred to a polyvinylidene difluoride (PVDF) membrane by electroblotting. The membrane was blocked for 30 min at room temperature in a blocking reagent (20 mM Tris, pH 7.4; 125 mM NaCl; 0.2% Tween 20; 5% non-fat dry milk; and 0.1% sodium azide) and then incubated for 2 h with anti-human OPG (1:2000, 0.53 μg/mL), RANKL (1:1000, 0.2 μg/mL), Ac-H3 (1:500, 0.2 μg/mL) and GAPDH (1:1000, 0.1 μg/mL) antibodies. Membranes were washed three times with TBST (10 mM Tris, pH 7.5; 100 mM NaCl, 0.1% Tween-20) for 10 min each, and then incubated with horse radish peroxidase (HRP)-labeled goat anti-mouse secondary antibody for 1 h. The membrane was then washed 4 times with TBST. Finally, the immunoreactive bands were developed by enhanced chemiluminescence (ECL) reagent and visualized on Fuji X-ray film. 

### 4.7. Effect of Butyrate on OPG, RANKL, 8-Isoprostane, Pro-Collagen I, MMP-2, Osteopontin, Osteonectin and Osteocalcin Secretion and the ALP activity of MG-63 Cells

Cells were treated with butyrate as above (Section 2.2). The culture medium was collected before MTT assay and used for the enzyme-linked immunosorbant assay (ELISA) of OPG, RANKL, procollagen I, MMP-2, osteopontin, osteonectin, osteocalcin and 8-isoprostane levels according to the instructions of the ELISA kit. For the assay of ALP activity, MG-63 cells were incubated in medium containing various concentrations of butyrate for 3 days. ALP activity was analyzed by following the procedures of ALP staining kits from Sigma-Aldrich as described previously [61,62].

### 4.8. Statistical Analysis 

Three or more independent experiments were performed. The results were statistically analyzed by paired Students *t*-test. A *p* value < 0.05 was regarded as indicating a statistically significant difference between groups.

## Figures and Tables

**Table 1:** Induction of apoptosis and necrosis of MG63 cells by various concentrations of butyrate as analyzed by PI and annexin V flow cytometry (*n* = 4). No statistically significant difference was noted between groups. LL (lower left).

Quadrant	Control	8 mM Butyrate	16 mM Butyrate	24 mM Butyrate
UR	0.85 ± 0.10	1.25 ± 0.19	1.28 ± 0.2	1.43 ± 0.40
UL	4.19 ± 1.12	4.19 ± 0.57	4.24 ± 0.51	4.79 ± 0.33
LR	0.41 ± 0.06	0.86 ± 0.21	1.05 ± 0.30	0.75 ± 0.24
LL	94.54 ± 1.05	93.69 ± 0.31	93.41 ± 0.18	93.02 ± 0.36
